# Biomonitoring and Biomarkers: Exposure Assessment Will Never Be the Same

**DOI:** 10.1289/ehp.8755

**Published:** 2006-04-26

**Authors:** Dennis Paustenbach, David Galbraith

**Affiliations:** ChemRisk Inc., San Francisco, California, USA

**Keywords:** biomarker, biomonitoring, exposure assessment, risk assessment

## Abstract

Using modern analytical technology, it is now possible to measure almost any chemical present in our bodies. The future role of classical exposure assessment will perhaps be marginalized because biomonitoring programs can directly measure the concentration of chemicals that are present in biologic matrices. Although the concentration of chemicals in the environment will continue to be measured and related to exposure parameters, the prioritization of the national environmental agenda will be dictated by biomonitoring. Recent biomonitoring studies have examined the levels of > 200 chemicals. Biomonitoring data, by themselves, are not informative in helping consumers understand their individual health risk. A major challenge facing those who conduct biomonitoring programs is how to best communicate the information to the public. In this article, we review benefits and challenges, along with select results from the Centers for Disease Control and Prevention’s 2005 *National Report on Human Exposure to Environmental Chemicals*. We recommend that these data be carefully interpreted, with the goal of establishing baseline exposure information, rather than creating surrogates for conclusions about human health risk.

We are all exposed to thousands of natural and man-made chemicals every day. Man-made and naturally occurring chemicals are present in the air, ingested food and water, the workplace, and a number of consumer products. The exposures that animals, plants, and humans experience can be quite complex, coming from a variety of potential sources (Figure 1). The Centers for Disease Control and Prevention (CDC), through its ongoing National Health and Nutrition Examination Survey (NHANES) project, has made it increasingly clear that each of us has ingested, inhaled, or absorbed a variety of these chemicals, with many widely measured in blood or urine in regions across the United States (CDC 2005). Sampling and measuring these chemicals and other agents is called “biomonitoring” (American Industrial Hygiene Association 2004; DeCaprio 1997; Kamrin 2004; Metcalf and Orloff 2004). As a result of well-conducted biomonitoring studies, we can obtain a picture of the amount of a chemical or agent actually absorbed into the human body. Because of much-improved tools and techniques discovered in the field of analytical chemistry, it is now possible to detect extraordinarily low concentrations of environmental chemicals in human tissue (parts per trillion and parts per quadrillion). Based on information from both toxicology and epidemiology studies, most chemicals that are currently detected in biomonitoring programs of the general population are not expected to produce adverse health effects at the levels experienced in our society, although for many chemicals more research is necessary to affirm this notion (CDC 2003, 2005).

Data from biomonitoring investigations are becoming more widely available and are frequently considered “newsworthy,” greatly heightening public interest in these investigations (CDC 2005; San Mateo County Environmental Health 2005; Williams 2005). However, these data are often presented without proper context, which can lead people to the understandable but erroneous conclusion that the low levels of chemicals found in our tissues are harmful, simply by virtue of their presence. In other words, detection does not necessarily equal risk. Reporting data without some type of explanation can frequently generate confusion and unnecessary anxiety. For a few chemicals that are sometimes monitored in manufacturing workers, we can properly characterize the meaning of the data. However, for most of the > 200 chemicals that are now being monitored by the CDC and other organizations, relating a chemical exposure to a measurable health risk is problematic, and we simply need to do more research to properly inform the public (Keshava et al. 2005; Perera et al. 2005).

A relatively recent development in the evolution of biomonitoring has been the attempt to assess exposures of the general population to chemicals found in the environment (“environmental” or “population biomonitoring”) (CDC 2003). Over time, one can envision conducting biomonitoring of indicators of biologic susceptibility to certain industrial or pharmaceutical agents.

The selection of which chemicals to study has been a complex and hotly debated topic. The CDC has identified a number of important variables that influence their nominating process:

- Evidence of exposure in a U.S. population
- The presence and significance of health effects after a given level of exposure
- Desire to track public health initiatives to reduce exposure to a given agent
- Existing method for accurately measuring biologically relevant concentrations of the chemical
- Sufficient tissue specimens, in particular, blood and/or urine samples
- Cost-effectiveness.

For their most recent report, the CDC obtained nominations for hundreds of chemicals and used a scoring process (CDC 2005). In transitioning from exposure estimations to direct biomonitoring, however, there will certainly be some obstacles. First, there are few data regarding animal levels of chemical agents. A fair comparison of animal data with human data would seem to demand that we also obtain internal levels in animals. Also, extrapolating human measurements from one point in time to chronic or even lifetime exposures may make sense for agents with extremely long half-lives, but for most agents one needs a series of data points to accurately estimate chronic exposures and any associated health risks.

## Biologic Media Commonly Analyzed

The new generation of analytical devices has made it possible to search for and detect in biologic tissue essentially any substance found in the environment. For practical and ethical considerations, only blood and urine have generally been collected. Additionally, factors such as exposure route, metabolism, and the volatility of the chemical often dictate which types of human samples can be most easily analyzed (Needham et al 2005). The benefits and shortcomings of different sample types are described below.

### Whole blood/serum

Blood is commonly used, largely because it is relatively easy to collect, is regularly replenished, and is one of the pathways through which most chemicals and their metabolites travel within the body. A disadvantage of using blood is that some people are opposed to having their blood drawn because of the invasiveness of phlebotomy or their religious or cultural beliefs. Additionally, because the life span of a red blood cell is only around 90–120 days, past exposures to some chemicals may be underestimated. This is especially true for those chemicals that directly interact with the red blood cell such as hexavalent chromium.

### Urine

The collection of urine samples is often the preferred method for biomonitoring programs, but for most chemicals it is not a reliable indicator of exposure. Only a few parent compounds are excreted in the urine, so typically one must test for excreted metabolites. Sometimes, the chemicals that one might want to study can also appear in the urine of “unexposed” persons, such as benzene in cigarette smokers. Toxicokinetics may also be important because in many cases the chemical of interest is excreted slowly over the course of hours or days after exposure, making it necessary to collect a 24-hr urine sample rather than a single sample. Timing of sample collection may also be important (Kissel et al. 2005). The primary advantage of using urine is that it is noninvasive and therefore can be an attractive methodology for conducting some large-scale studies.

### Adipose tissue (fat)

Adipose tissue studies have, in the past, been used to study fat-soluble chemicals. However, since about 1990, it has been possible to obtain very similar information about exposure from the fat-soluble fraction of blood. For this reason, very few adipose tissue samples are now collected.

### Hair

Hair analysis techniques have improved in recent years because of increasing use by forensic scientists, especially in studying drugs of abuse. Hair analysis has also been applied to the study of certain environmental chemicals such as mercury, arsenic, and manganese. Of course, it is important to distinguish chemicals that are internally deposited in hair from those that land on the hair and are absorbed into it.

In addition, it is important that a careful protocol be followed during the sampling and analysis of human hair due to differing chemical concentrations along the length of the hair. Few studies have measured occupational or environmental exposure and then compared it with results from hair analysis. By and large, most investigators use hair analysis only as a screening tool. A recent scientific panel that evaluated the utility of hair sampling for assessing exposure to chemicals routinely found in the ambient environment concluded that the method is too inconsistent to be relied upon, with the possible exceptions of methylmercury and arsenic (Harkins and Susten 2003).

### Breast milk

Breast milk can often provide significant information about fat-soluble chemicals in the environment. Nearly all of the industrially significant fat-soluble chemicals, such as polychlorinated biphenyls (PCBs), brominated flame retardants [poly-brominated diphenyl ethers (PBDEs)], and dioxins/furans can be measured in breast milk. Samples of breast milk are relatively easy to collect and may reflect historical exposures to lipid-soluble chemicals found in the environment. Diet plays a significant role, with many of the chemicals in breast milk arising from a mother’s consumption of fish, meat, or dairy products. These can contain small amounts of persistent, lipid-soluble chemicals coming from deposition of dust, vapors, or ash emitted from incinerators and nearly every form of combustion, including automobiles and furnaces.

Exposures to chemicals around the home and in processed foods are also easily measured in breast milk (Kamrin 2003). One can postulate that the breast-feeding infant is at the top of this exposure chain. Because the infant has such a small comparative body mass and breast milk can compose a significant fraction of the diet for the first year of life or longer, there has been considerable interest in understanding the potential effects of these chemicals in the developing infant. However, no documented adverse effects from infant exposures to typical levels of environmental chemicals in breast milk have yet been identified. A recent workshop explored a number of issues surrounding biomonitoring, breast milk, and risk assessment (LaKind et al. 2005).

### Saliva and sputum

Saliva testing has not been widely used, but it may hold promise as a future noninvasive method for determining exposure to some chemicals and has been used by some laboratories to measure concentrations of naturally occurring steroid hormones. Researchers have explored its use as a monitor for therapeutic drug levels, with mixed results (Madsen et al. 2004; Miles et al. 2003; Shiran et al. 2005) as well as for documenting exposures to lead, pesticides, and cigarette smoke (Denovan et al. 2000; Lu et al. 2003; Timchalk et al. 2004, 2006; Woodward et al. 2005). Saliva was first used as early as the 1950s in assessing the exposure to some chemicals in the workplace.

Sputum, on the other hand, has been used in the past, but it has proved to be a much less reliable data source, largely because of the inconsistent quality of individual specimens. The high variability in specimen quality can be circumvented with invasive procedures, such as transtracheal aspirates or suctioning material through a bronchoscope or endotracheal tube, but these techniques are not likely to be chosen as screening techniques for the broader population. Previous studies have evaluated sputum for tracking exposure to asbestos, heavy metals, and other inhalational agents (Josyula et al. 2006; Lemiere et al. 2001; McDonald et al. 1992; Paris et al. 2002; Park et al. 2003).

### Semen

Semen has not yet been widely used, but it is believed by some experts that semen may turn out to be a better test medium than many other bodily fluids currently being evaluated.

### Exhaled air

This method is most applicable in the study of volatile chemicals and some gases. Gustafsson et al. (1991) described the measurement of nitric oxide in exhaled air, a technique that now is used as a noninvasive means of determining airway inflammation in certain populations (American Thoracic Society 2005). The noninvasive character and reasonable expense generally associated with the analysis of expired air make this an attractive option for a select subset of chemicals.

## Sources of These Chemicals

The chemicals detected by biomonitoring generally come from three types of sources: anthropogenic or man-made processes, normal biologic processes, and naturally occurring chemicals in food.

### Anthropogenic (relating to the activity of humans)

People may be exposed to man-made chemicals as a result of their daily activities, including working, using consumer products, and eating.

Persons working in certain occupations have an increased likelihood for substantial exposure to chemicals. Careful study of these individuals has been invaluable in allowing scientists to better understand the hazards posed by these agents (Lan et al. 2004; Paustenbach et al. 1992). For example, as a result of workplace exposures, we know that elevated concentrations of certain metals can increase the risk for a number of acute illnesses such as neuropathies, kidney damage, and liver damage. Since the organic chemical revolution began in the 1920s, numerous chemicals now in our environment possess a long biologic half-life, and most of these can be easily measured today.

Many consumer products contain lipid-soluble chemicals, and these are readily detectable because of their sequestration in fatty tissues. Several of these chemicals are also present in foods, which can make it difficult to attribute the exposure to a particular source.

In addition to indirectly influencing our foods and the workplace, more generally the air we breathe is a source of chemical exposure. Respiratory effects of chemicals have attracted significant attention over the past few decades, resulting in many regulatory initiatives (e.g., the Clean Air Act of 1990) with widespread societal effects (reduced vehicle emissions, burning restrictions, propellants and other additives in consumer products, requirements for cleaner fuels).

### Normal biologic processes

Background levels of many chemicals in blood or urine occur as a result of normal biologic function. For example, chemicals such as formaldehyde and methanol are produced as a consequence of normal human metabolism but can also reflect exposure to industrial solvents, wood products, or decomposition of biologic wastes. When such duplicative sources are possible, making the association of the laboratory data with exposure to a specific man-made agent can be very difficult (Dettmer and Hammock 2004).

### Chemicals in food

Foods generally represent the largest source for chemicals found in our bodies. Indeed, the World Health Organization (WHO) believes that most chronic health conditions can be attributed to past and current exposure to chemicals in the foods we eat (WHO 2005). Meat from grazing animals contains chemicals present in the plants they eat. These plants often contain chemicals that came from the deposition of particles released from combustion. In addition, vegetables contain naturally occurring chemicals that bind to the aryl hydrocarbon receptor (the same receptor necessary to initiate dioxin toxicity) at a level > 45,000 times greater than the dose of polychlorinated dibenzo-*p*-dioxins (PCDDs), polychlorinated dibenzofurans (PCDFs), and PCBs in a typical American diet (Connor et al. 2005; Finley et al. 2003), although the half-lives of these naturally occurring chemicals tend to be much shorter. Similar results were reported by Jeuken et al. (2003) in their study of dietary herbal supplements and other common food products. Finally, sometimes chemicals are added during the processing of foods that then show up in our diet or water supply.

## Interpretation of Biomonitoring Data

Currently, the standard method for estimating exposure is to use mathematical models to estimate the concentration of a chemical in various media to which persons were thought to have been exposed. To perform the calculation, researchers frequently collect information on soil, water, air, dust, and food concentrations and couple this with data on human behaviors (how much air one breathes or food one eats). All of the input parameters have some degree of variability, potentially increasing the degree of uncertainty in the answers obtained. If properly conducted, this approach may yield a reasonably accurate estimate of the absorbed dose. However, without some confirmation of the exposure estimates through an approved method of biologic sampling, it is often not possible to validate the accuracy of the exposure assessment.

By measuring the “body burden,” or total amount of a chemical or its metabolites in the human body, one can obtain direct evidence of actual human exposure (Sexton et al. 2004). Unlike the data in other types of risk assessments, biomonitoring requires no assumptions regarding exposure parameters such as ingestion or inhalation rate, bioavail-ability, or frequency of exposure. It can also provide specific information on an individual regarding his or her particular set of exposures. For example, dioxin levels in blood can be a reflection of exposures that occurred over the prior 20–25 years. It is usually not possible, however, to know whether the total dioxin levels are primarily due to ingestion of fish, meat, or dairy products over the previous few months, or whether they are due to chronic exposure from living near an industrial facility. However, if one understands the elimination rate of the specific chemical being measured and can estimate the date(s) of last exposure, then it may be possible to approximate the typical daily exposure that occurred in the past and relate it to current results (Aylward et al. 1996).

It is also important to recognize that exposure to a potentially hazardous substance does not necessarily result in clinical disease or toxicity. For example, few persons are worried about the risk of ingesting two aspirin tablets (650 mg) per day, but most know that there can be serious adverse health effects associated with ingesting a much higher dose, say, ≥ 20 aspirin pills per day. Most, if not all, toxicity studies provide information about the relationship of daily dose and an adverse health effect. In order to correlate toxicity data with biomonitoring data, an estimate of daily dose must be performed. If that can be reasonably calculated, it may then be possible to estimate the likelihood of an adverse health effect and the severity of that effect.

One of the most significant challenges in biologic monitoring is characterizing the relevance of the data. Detection alone means virtually nothing because very low concentrations of dozens of chemicals have been detected in our environment for at least the past 50 years. What is important is to discover whether or not these trace concentrations are actually exerting some measurable effect. This is certainly a complicated question because, on average, Americans are living longer than ever before and have much higher expectations regarding their quality of life in the later years. At the same time, however, the incidence of certain cancers such as prostate cancer, breast cancer, and melanoma is increasing (Edwards et al. 2005), and chronic diseases such as obesity and diabetes are also on the rise. By comparing community blood levels with appropriate animal or human studies examining the hazards posed by a specific chemical, one can reasonably determine whether there are increased health risks associated with environmental exposure. It is also important to recognize that some diseases (e.g., prostate cancer or senile dementia) may be showing an increased incidence simply because people are living longer.

Other factors to be considered when interpreting biomonitoring data include toxicologic factors, such as validity of the toxicologic or epidemiologic study, toxicokinetic considerations, adequacy of the sample size, reproducibility of the sample, and presence of confounding elements, and exposure factors, such as time since last exposure, average dose received, and frequency of exposures. In certain cases, a particular biomonitor may have been widely used for many years, and the aforementioned factors may have been accounted for and validated by a reputable scientific body. Thus far, only a few biomarkers of environmental chemicals have been through this level of scrutiny and are considered useful in evaluating the health of persons in the community. Others, such as phenol in urine due to exposure to benzene, dioxins in blood, urinary mercury, and a number of others, have been useful in studying workers. Because most criteria for biologic monitoring are based on extrapolations of animal or human data where exposures were at very high concentrations, it is often not possible to predict that an adverse effect will occur in a specific individual or group of individuals who have a lower concentration of a biomarker. Ideally, in future toxicologic studies, blood levels will be routinely measured and compared with the delivered dose to facilitate the interpretation of biomonitoring studies in humans. One important challenge facing scientists with respect to the use of biomonitoring data is to ensure that appropriate normative values are available to interpret test results. Background levels, or the typical range of biomarker concentrations expected in the general population, are fundamental for this analysis. Some of these data have been compiled by NHANES (CDC 2003, 2005).

The National Center for Environmental Health (Atlanta, GA) has measured concentrations of > 100 environmental chemicals in the blood of the general population. These are frequently reported as means and various upper percentiles (e.g., the 95th percentile). Although determining the percentile ranking of an individual’s biomonitoring results does not necessarily suggest that an adverse effect is expected, a measurement markedly above the 95th or 99th percentile can alert scientists that the individual is experiencing some unique exposure that may be worth documenting and characterizing. To understand the health risk, however, one must compare this kind of information with data collected in toxicologic and/or epidemiologic studies. In a summary of an interdisciplinary panel in 2004, Bates et al. (2005) provide an excellent review of biomonitoring study design and many of the issues around interpreting biomonitoring data.

## Limitations

### Information regarding exposure

The presence of a biomarker does not reveal the source or route of exposure. When there is only one potential source of exposure to the chemical, for example, cobalt in the diet, the challenge may be minimal. However, in the case of dioxins, there can be many different sources of exposure, and sorting out the actual source will generally be difficult and sometimes impossible.

Appreciation of the wide variability in chemical half-lives is also important. Some chemicals, such as dioxins and PCBs, are fat soluble and resistant to degradation. These chemicals have biologic half-lives measured in years (Table 1), which makes it feasible to detect significant exposures sometimes decades after they occurred. Conversely, many metals, volatile organic compounds, and water-soluble compounds are rapidly eliminated from the body, with half-lives of a few hours to a few days. After exposure to such a chemical, it is necessary to promptly collect the biologic sample before the chemical has been excreted. This limits the feasibility of biomonitoring studies for these types of chemicals. If the exposures are intermittent and the biomarker is short-lived, it is often difficult to collect suitable samples for biomonitoring. For example, one study attempted to look at the chemical toluene diisocyanate (TDI) in a population with intermittent exposures (Metcalf and Orloff 2004). Because of TDI’s short half-life, biologic samples were not useful. Instead, investigators measured antibodies to TDI, which have a longer presence in the body. In some cases, where chemicals are known to have a short half-life and are widely measurable in the population, this indicates a frequent, possibly continuous exposure to the chemical of interest (e.g., cotinine or some phthalates). For every chemical involved in biomonitoring studies, it is necessary to give considerable thought to the toxicokinetics before one tries to interpret the significance of the data obtained.

### Health consequences of chemical exposure

**Figure 2:**
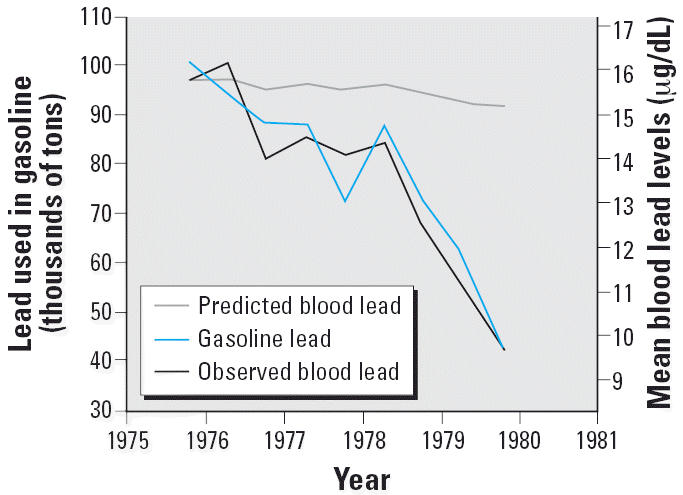
Sharp decline in human lead levels in the United States, as demonstrated in the NHANES III data set, compared with the levels predicted by U.S. EPA calculations (CDC 2005; Needham 2005). This decline continued for two decades.

For a few chemicals, such as lead, human data from occupational and other clinical studies allow us to identify body burdens of a chemical that may result in an adverse health effect. This information can be used to justify reduction or removal of a particular chemical from consumer products or cleanup and monitoring of affected environments. Figure 2 demonstrates the remarkable impact that regulatory actions and public awareness have had on lead levels in our society, after adequate scientific recognition of the health effects of lead on humans. For other chemicals, there may be a suspicion of adverse effects, found perhaps in animal studies or in case reports, but the assembled evidence is not yet compelling. For most chemicals, however, we do not have adequate human data to be certain about health effects, particularly at very low chemical concentrations. In addition, most environmental exposures involve multiple substances, and attributing cause to a single chemical can often be difficult. These complexities should be revealed to groups being studied before they are allowed to give consent to participate in an investigation.

## Benefits of Biomonitoring Programs

**Figure 3:**
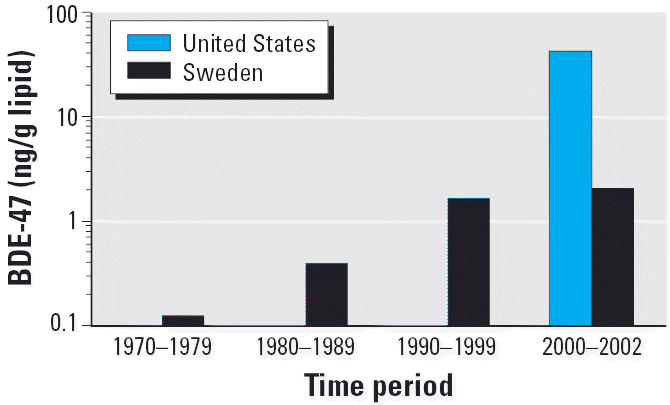
Trend of increasing concentrations of BDE-47 (2,2′,4,4′-tetraBDE), one of the PBDE congeners most frequently detected in breast milk samples collected in Sweden and the United States. Based on data from Meironyté et al. (1999) and Schecter et al. (2003).

Perhaps the primary benefit that can be obtained from biomonitoring is the identification of long-term trends in the population. For example, it has been observed that blood levels of PBDEs have increased in the general population over the past 10 years in the United States [Agency for Toxic Substances and Disease Registry (ATSDR) 2004; Stokstad 2004]. PBDE levels have also increased in samples of human breast milk collected in Sweden (Figure 3). However, other studies have shown that the total body burden of organohalogen compounds has actually been decreasing over time (Meironyté et al. 1999; Schecter et al. 2003). Comparing these observations encourages an understanding of why the levels of PBDEs appear to be increasing while the broader category of organohalogens appears to be decreasing. Regulations proposing the banning of PBDEs in consumer products exist in some European countries, based primarily on the results of biomonitoring studies. Proper interpretation of these data, however, requires careful consideration of dose, duration of exposure, and toxicity. After performing appropriate studies, public health policy makers and scientists then need to decide whether reduction of chemical release into the environment is necessary, and whether the relatively higher U.S. levels of PBDEs in breast milk noted in Figure 3 constitute a true health hazard. Often, only one or two sources may account for the vast majority of the exposure, and sometimes these sources can be significantly reduced or eliminated.

Another potential benefit of a national biomonitoring program is identifying those geographic locations where people have much different body burdens than the general population. For example, if the inhabitants of a particular coastal area have a higher than expected concentration of PCBs, this might suggest that there is a local source of exposure to PCBs that is extraordinary. In this scenario, scientists might conduct an evaluation of meat, fish, and dairy products to determine whether diet is a significant source for the chemical. If the dietary intake is found to be within normal ranges, then one might progress to a study examining the possible historical or current industrial sources of PCBs. After scientists have obtained a good understanding of the variability of typical background concentrations in the nation, biomonitoring could identify “hot spots” that deserve attention.

Biomonitoring data might also reveal unusually elevated releases of environmental chemicals. For example, one might identify a specific community where half of the inhabitants have concentrations of a persistent chemical in their blood that is at the 95th percentile of the national levels. This would suggest a local source, and if some consistent adverse effects were seen in this group, the population could be studied to further advance our understanding of the chemical and its potential impact on society. Only a few chemicals in the general population have been studied in this way, such as lead and arsenic, which led to guidelines regarding the possible health risk associated with certain concentrations found in blood or urine.

**Figure 4:**
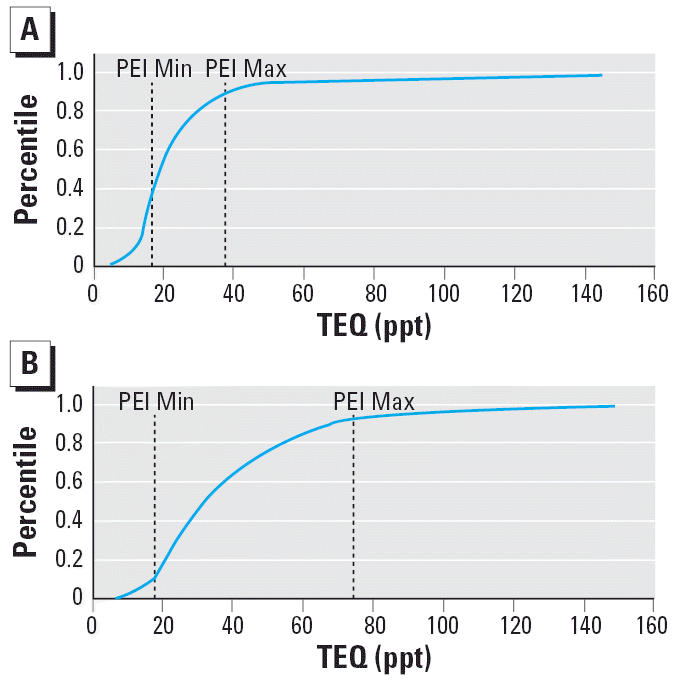
Toxic equivalent (TEQ) levels of PCDDs/ PCDFs and select PCBs (congeners 81, 126, and 169) measured in 45–59 year (*A*; PEI range = 36th–89th percentile of age-specific NHANES) and ≥ 60 year (*B*; PEI range = 12th–93rd percentile of age-specific NHANES) age groups from the NHANES data set (CDC 2005). Abbreviations: Max, maximum; Min, minimum; PEI, pilot exposure investigation. Note the higher levels found in the ≥60 year age group.

Properly conducted, biomonitoring could also help deliver valuable evidence for epidemiologic studies, which are frequently plagued by weak exposure data. Biomonitoring would provide unequivocal evidence of exposure and be able to yield quantitative information. The importance of certain factors, such as age group stratification, can provide insights into human exposures, particularly for chemicals that have long half-lives (Figure 4). More important, if the toxicokinetics and dermal absorption are possible to obtain, they can provide specific data quantifying the magnitude of exposure so that a dose–response relationship can be properly characterized. Having these data would greatly elevate the merit and possible impact of an epidemiologic study.

Biomonitoring can also reduce the uncertainty inherent within traditional exposure and risk assessments. In general, these assessments have relied on calculations that attempt to quantitatively account for intake of a chemical as a result of soil ingestion, drinking water, breathing the ambient air, ingesting foods, or exposure to house dust, based on estimates of concentrations of various chemicals in the specific media. Many also rely on experimental data from high-dose chemical exposure studies in animals when estimating the “safe” or tolerable dose and fail to account for a variety of uncertainty factors in inter-species variation. Biomonitoring data could eliminate or reduce much of this uncertainty in estimating risk because internal dose and response information would be directly available for a human population.

Establishing statistical trends with bio-monitoring data can aid in determining the progress of specific policy decisions to reduce or eliminate particular compounds in the environment or occupational setting. For example, blood lead levels in the United States have dropped > 90% with the elimination of lead as a gasoline additive (Figure 2); PCB levels have also consistently declined in the last 20 years as PCBs have been phased out by industry (Kamrin 2003; Metcalf and Orloff 2004). The same phenomenon has been observed with DDT (dichlorodiphenyltrichloroethane), chlordane, and heptachlor. Scientists had a sense that reductions of these chemicals in the general environment would lessen body burdens in our society, but the time frame over which this would occur and the magnitude of its decline would not have been clear were it not for biomonitoring programs.

## How Biomonitoring Will Cause Change

Improving and protecting human health have always been important ideals. The tools that scientists have had at their disposal to measure toxicants in our environment, understand their possible impact on biochemical processes, and evaluate their cumulative effects on people, animals, water, air, food, and soils have evolved rapidly, especially in the last decade. Biomonitoring also allows insight into what a specific individual has experienced, and how his or her exposures may lead to an increased risk of morbidity or mortality.

The penetration of biomonitoring into our social fabric has generated a wholesale shift in focus from the environment as a model for human exposure—a method that requires approximations regarding concentration, length of exposure, frequency of exposure, “susceptibility,” absorption, distribution, metabolism, and excretion—to a focus on the individual. Scientists are now able to understand how exposure translates into absorption of a particular substance into various tissues of the body. As we have observed with the NHANES initiative, scientists are now able to measure hundreds of chemicals in members of our society and compare the results across a variety of geographic regions, age brackets, and personal habits. We can subdivide the data by sex, by racial background, by socioeconomic group—our limits increasingly have become not mechanical, but instead pragmatic. That is, which substances are considered most likely to be linked to disease or dysfunction? Who should we examine? How much will it cost to perform the study? How do we go about rectifying the problem if we determine that one exists?

With scientific or technologic advancement comes responsibility. As scientists, we should have a commitment to not just providing data but also to providing perspective. Our viewpoints need to help shape reasonable decision making by our leaders and avoid alarmist interpretations of data by those who would seek to distort for their own ends. On the other hand, we must also be attentive to significant health effects that can be meaningfully related to a given exposure or set of exposures.

Before the “tide” of measuring chemicals hits the public and an increasingly anxious citizenry creates a demand for home mass spectrometers, our public health leaders and governmental agencies have a duty to inform, to help people appreciate that our ability to detect far surpasses our ability to detect meaning. To properly ascertain the risk of a given biologic level of a chemical will require well-controlled studies that demand resources, time, and careful planning. In an era of cost containment at the federal, state, and local levels, the process of committing our scarce resources in the most effective manner possible will be of paramount importance.

## Conclusion

Biomonitoring programs are likely to produce a sea of change with respect to increasing the awareness of the public to the presence of chemicals in our diet and environment. In addition, it is about to revolutionize, and perhaps marginalize, the importance of much of environmental monitoring as a screening tool. It can be expected that biomonitoring will become the first indicator of concern and that environmental measurements will be used to identify the source of the contamination.

Currently, data are being shared in the press and on the Internet without discussion of their significance to human health. Indeed, characterizing the significance of these data to the overall healthfulness of the public is a daunting task that no organization appears ready to tackle. In many cases, until a baseline data set is well established, there is not a great deal that can be safely concluded without conducting a fairly careful risk assessment. It is probably safe to say that virtually all scientists agree that there needs to be a greater understanding of the potential risks to human health posed by the various chemicals to which Americans are exposed on a daily basis. It is important, however, to use biomonitoring as a tool to help guide our social and scientific leaders to make intelligent decisions rather than as a method for producing fear. Thus, the scientific and regulatory communities need to begin to initiate programs for communicating effectively with the public about these data.

Biomonitoring, in its broadest sense, offers great opportunities for identifying persons who are unknowingly exposed to both naturally occurring and industrial chemicals. Currently, because most biomonitoring information is designed to characterize background levels of a subset of chemicals in the U.S. population, those conducting these programs must be aware of the unintended consequences of sharing data that frequently are insufficient to inform us of the presence of increased risk. In our view, scientists have a responsibility not only to do the hard work of relating concentration with the toxicology and epidemiology data but also to begin conducting risk–risk tradeoff analyses, an exercise that has been well developed over the past 20 years. These techniques offer the opportunity to provide the public with the means to sensibly decide how to balance the presence of chemicals in the environment versus the possible risks. It is time for those of us in public health to do a more thorough job of using all of our knowledge about risk assessment and risk benefit in helping to inform everyone.

## Figures and Tables

**Figure 1:**
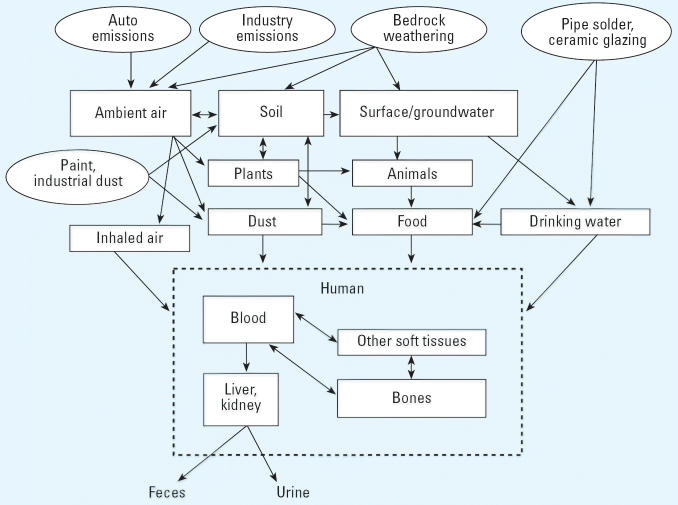
Pathways of elemental lead exposure to biologic media illustrating the complex relationship between chemical release, exposure, and biologic media [modified from U.S. Environmental Protection Agency (EPA) 1998].

**Table 1:** Half-lives of some exposure biomarkers.

Half-life	Chemical	Indicator	Sample
Very short			
2.5 hr	Benzene	Benzene	Blood and exhaled air
3.5 hr		Phenol	Urine
5 hr	Carbon monoxide	Carboxyhemoglobin	Blood
Short			
5 hr	Styrene	Mandelic acid	Urine
14 hr	*n*-Hexane	2,5-Hexanedione	Urine
18 hr	Polycyclic hydrocarbons	Pyrenol	Urine
96 hr	Perchloroethylene	Perchloroethylene	Blood and exhaled air
Long			
18 days	Mercury	Mercury	Blood and urine
30 days	Lead	Lead	Blood
100 days	Cadmium	Cadmium	Blood
Very long			
2 years	Hexachlorobenzene	Hexachlorobenzene	Serum
5 years	Lead	Lead	Bones
7 years	2,3,7,8-TCDD	2,3,7,8-TCDD	Blood
> 10 years	Cadmium	Cadmium	Urine

2,3,7,8-TCDD, 2,3,7,8-tetrachlorodibenzo-*p*-dioxin. Modified from Santamaria (2005).
